# A finite element analysis model to support ligamentum teres function

**DOI:** 10.1093/jhps/hnaf017

**Published:** 2025-04-18

**Authors:** Yongni Zhang, Jianing Wang, Linxia Gu, Hal David Martin, RobRoy L Martin

**Affiliations:** Duquesne-China Health Institute, Duquesne University, 600 Forbes Ave, Pittsburgh, PA, 15282, USA; Biomedical Engineering and Science, Florida Institute of Technology, 150 W University Blvd, Melbourne, FL 32901, USA; Biomedical Engineering and Science, Florida Institute of Technology, 150 W University Blvd, Melbourne, FL 32901, USA; Hip Presentation Center, Baylor University Medical Center, 3500 Gaston Ave, Dallas, TX, 75246, USA; Department of Physical Therapy, Duquesne University, 600 Forbes Ave, Pittsburgh, PA 15282, USA; UPMC Center for Sports Medicine, 3200 S Water St, Pittsburgh, PA, 15203, USA

## Abstract

The function of the ligamentum teres (LT) remains debated, particularly its role in limiting motion. The aim of this study was to use finite element analysis to assess LT stress during hip movements, which included external rotation with flexion. A 3D model of the hip joint, including the femoral head and LT, was constructed from magnetic resonance imaging data using 3D Slicer. The models were imported into Ansys SpaceClaim 2022R1 for refinement and assembly. The von Mises stress in the LT was extracted during six hip movements: external rotation, internal rotation, abduction, adduction, flexion, and extension. LT stress response was also extracted during external rotation at hip flexion angles of 0°, 30°, 60°, and 90°. The results found there was a sharper increase in LT stress during movements involving hip external rotation, internal rotation, abduction, and adduction when compared to movements in flexion and extension. External rotation in larger hip flexion angles resulted in greater LT stress, with the highest stress observed at 90° flexion. These findings help to support the LT’s role as a rotational stabilizer in the frontal and transverse planes wrapping around the femoral head to act as a sling. Additionally, the increased stress during external rotation at greater degrees of hip flexion suggests an enhanced role for the LT in hip stability as flexion increases. These results add as a proof of concept in that the LT is under stress during hip movements and has a potential role in stabilizing the hip joint.

## Introduction

The ligamentum teres (LT) is a unique structure within the hip joint and its function remains a topic of debate. The prevalence of LT pathology has ranged between 43% and 51% in consecutive hip arthroscopies, with individuals experiencing LT tears reporting significantly lower functional outcome scores compared to those without tears [1, 2]. A ball and string model has been used to describe the function of the LT with it wrapping around the femoral head to act as a sling during frontal (coronal) and transverse (axial) plane movements [3–6]. Cadaver studies however have presented conflicting conclusions regarding the LT’s role in limiting external rotational [3, 7, 8]. This inconsistency in findings highlights the need for further investigation into the LT’s function in stabilizing the hip.

Non-traumatic LT tears are thought to occur as the ligament tries to maintain stability within supra-physiological range of motion, often seen in sport activities [9, 10]. Martin *et al*. [5] simulated the LT using a string model and found the combination of hip external rotation in 90° hip flexion and hip adduction in hip extension produced the greatest length change in their LT model. Cadaver studies found the LT to be a transverse plane rotational stabilizer, particularly in greater degrees of hip flexion [1, 3, 8]. These findings highlight the role the LT may have as a transverse plane rotation stabilizer, which could be particularly significant in individuals with microinstability and focal rotational laxity of the iliofemoral ligament [3, 11]. However, there has been variability in the results of cadaveric studies. While Martin *et al*. [3] found LT to play a significant role in limiting external rotation of the hip, Jo *et al*. [7] found that the LT provided minimal influence in restricting external rotation. Additionally, van Arkel *et al*. [8] found that the LT served as a secondary stabilizer in limiting external rotation, with a much smaller contribution compared to the lateral iliofemoral ligament. The differing conclusions of these three studies support the need for further study regarding the role the LT has in controlling external rotation of the hip under different flexion positions [3, 7, 8]. Additionally, there is a need to further investigate the role of LT in controlling movements in the transverse and frontal plane.

Finite element analysis (FEA) is a computational modeling technique that allows for a non-invasive study of the mechanical response of tissues in orthopedic research [12]. Studies have successfully used FEA to model ligament function in knee and ankle joints based on computed tomography (CT) and magnetic resonance imaging (MRI) image data [13–15]. Specifically, FEA can help understand the mechanisms of ligament injuries and predict stress distribution in ligaments under loading [12]. The aim of this study is to use FEA support the role the LT has in controlling frontal and transverse plane movements of the hip. It is hypothesized that stress in the LT will increase as hip rotation and abduction–adduction increase when compared to flexion–extension at similar range of motion values. Also, when specifically looking at external rotation, it is hypothesized that stress in the LT will increase as hip flexion increases.

## Materials and methods

The FEA modeling was done similar to that previously described [16]. In brief, a 3D model of the hip joint with the femoral head and LT was constructed from a MRI data using 3D slicer (version 5.6.2, https://www.slicer.org/) from a 18-year-old male with a normal LT. These models were imported into Ansys SpaceClaim 2022R1 for further refinement and assembly. The LT was refined to ensure smooth interaction with femoral head structures. In order to simulate hip joint dynamics, the femoral head was modeled as a rigid body. The LT was treated as a flexible structure with Neo-Hookean hyperelastic properties, characterized by an initial shear modulus (µ) of 6.5 Mpa and an incompressibility parameter (D1) of 6.16 × 10⁻⁴/Mpa.

The contact between the LT and the femoral head was defined as bonded, assuming no separation during hip movement. A fixed boundary condition was applied to the free end of the LT. Six hip movements: external rotation, internal rotation, abduction, adduction, flexion, and extension were applied to the hip joint, and the von Mises stress in the LT were extracted to evaluate stress responses in the LT. Additionally, external rotation of the hip joint was applied in flexion angles of 0°, 30°, 60°, and 90° with maximum von Mises stress in the LT extracted during external rotation. Supplementary videos demonstrate three different simulations: (A) Flexion from 0° to 90° combined with external rotation, (B) Internal rotation at 30° to external rotation at 40°, and (C) Abduction at 45° to adduction at 30°.

## Result

**Figure 1.**
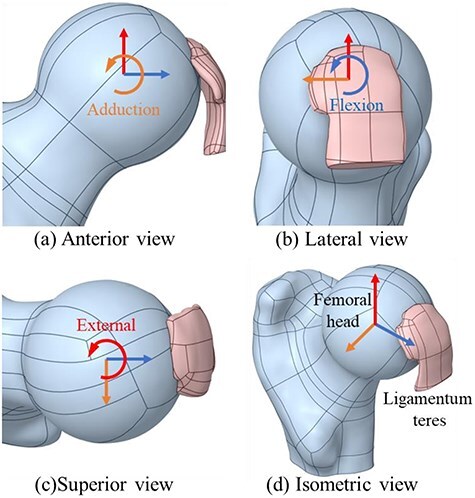
Finite element analysis model. Illustrations of the femoral head and LT with rotational movements of the femoral head: (a) anterior view; (b) lateral view; (c) superior view; and (d) isometric view.

**Figure 2.**
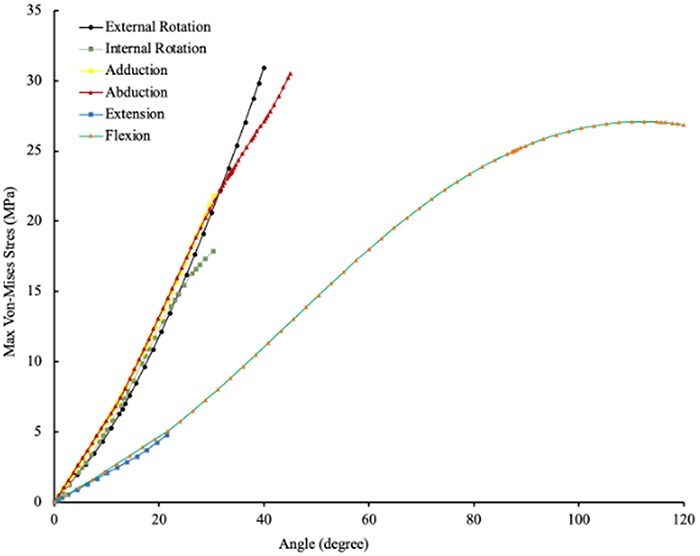
Stress in the LT across six hip movements.

**Figure 3.**
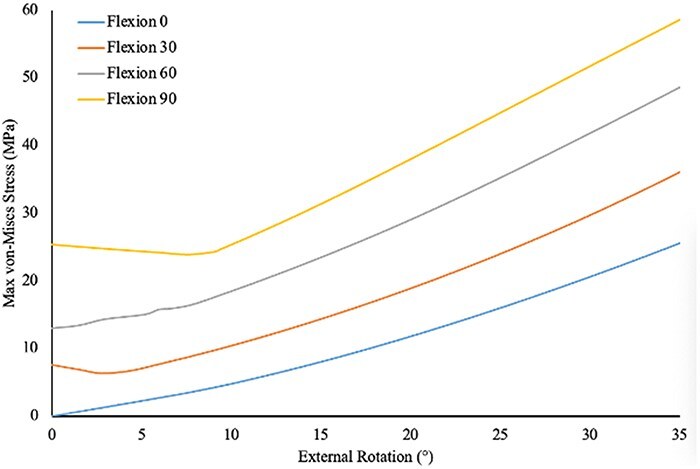
Stress in the LT during external rotation at hip flexion angles.

Figure 1 shows the 3D model of the hip joint including femoral head and LT. Figure 2 shows the stress in the LT during increasing ranges of motion (ROM) across six hip movements. Stress increased as follows: external rotation 0° to 40° = 0–30.9 Mpa; internal rotation 0° to 30° = 0–17.8 Mpa; abduction 0 to 45 = 0–30.5 Mpa; adduction 0° to 30° = 0–21.4 Mpa; extension from 0° to 20° = 0–4.9 Mpa; and flexion 0° to 120° = 0–26.8 Mpa. The results show that internal rotation, external rotation, abduction, and adduction have higher stress on LT compared to flexion and extension for the same angular values. There was also a sharper increase in LT stress during movements in frontal and transverse planes compared to movements in fontal plane. Figure 3 shows the stress in the LT during external rotation from 0° to 35° at hip flexion angles of 0°, 30°, 60°, and 90° with values increasing from 0 to 27 Mpa; 7.6 to 36.6 Mpa; at 60°, 13 to 51.3 Mpa; and 25.3 to 59 Mpa, respectively. The results show that higher flexion angles are associated with greater stress in the LT during external rotation.

## Discussion

This FEA model study found that the LT experiences a sharp increase in stress as range of motion increases in the transverse and frontal planes. These findings help to support the ball-and-string model with the LT functioning as a sling that wraps around the femoral head and tightening to add stability to the hip. Furthermore, the study found that increased hip flexion angles result in higher stress in the LT during external rotation supporting the potential role the LT adds to hip stability in hip flexion. These results add as a proof of concept in that the LT is under stress during hip movements and may have a potential role in stabilizing the hip joint, particularly with frontal and transverse plane movements.

The findings of this current study, with increasing stress in the LT with increasing hip external and internal range of motion, further support prior studies which have proposed the LT to functional as a rotational stabilizer in transverse planes [1]. Martin *et al*. [3] found an increase in hip internal and external rotation ROM in 18 positions of hip flexion–extension and abduction–adduction in a cadaveric study when comparing cut to intact LT conditions. The sharp increase stress in the LT seen with increasing range of motion in the transverse plane presented in Fig. 2 agree with those previous findings. In addition, this current study found that stress in the LT increased with external rotation as the degree of hip flexion increased (Fig. 3). Martin *et al*. [3] similarly found the most significant increase in external rotation ROM occurring at 90° or greater hip flexion. Martin *et al*. [3]. did not directly report stress on the LT during hip external rotation at different flexion positions, making direct comparisons with the current study challenging.

While the results of this current study are in agreement with some previous work [3], there are some inconsistencies. Van Arkel *et al*. [8] found that the LT limits external rotation when the hip is flexed beyond 60°. However, its contribution was noted to be minimal compared to the iliofemoral ligament. In contrast, Jo *et al*. [7] compared hip external ROM before and after arthroscopic sectioning of the LT and found that the LT prevented excessive external rotation at 60° and 90° of hip flexion but not at 110°. Those findings differ from Martin *et al*. [3] and current study. While both Jo *et al*. [7]. and Martin *et al*. [3] preserved the integrity of the hip capsule, discrepancies in their findings can be attributed to differences in experimental design and measurement approaches. Martin *et al*. [3] manually applied forces, while Jo *et al*.⁵ used a fixed torque of 4 Nm. Although the approach by Jo *et al*. [7] ensured consistency, the smaller forces may not have been sufficient to overcome any resistance of the capsule and therefore not fully assess the function of the LT. The hip capsule ligaments acting as stabilizers when hip external rotation at higher hip flexion angles is supported by Van Arkel *et al*. [8], who systematically resected the acetabular labrum and capsular ligaments to evaluate individual component contributions to external rotation. Furthermore, Jo *et al*. [7] used an electromagnetic motion tracking system, which is highly sensitive to small angular changes. This sensitivity enabled the detection of LT contributions at lower flexion angles (60°–90°), whereas Martin *et al*. [3] used a goniometer with potentially larger measurement error. However, Martin *et al*. [3] applied a stricter significance threshold (*P* < .0014), which resulted in the 60° hip flexion data (*P* = .006) being reported as non-significant. In contrast, Jo *et al*. used a significance threshold of *P* < .05, suggesting potential consistency between the two studies in identifying the contribution of the LT to hip stabilization at mid-range flexion angles.

Unique signs and symptoms of LT pathology are challenging to identify, as LT pathology rarely occurs in isolation [1]. Among patients undergoing hip arthroscopy, the prevalence of LT pathology is reported to range from 30% to 90% [1, 17]. Activities requiring extreme ranges of motion (e.g. ballet, gymnastics, martial arts), as well as conditions such as femoroacetabular impingement syndrome (FAIS), labral tears, labral degeneration, loose bodies, chondral damage, and articular cartilage degeneration, are frequently associated with LT pathology [1, 9, 17–20]. The high prevalence of LT tearing in individuals undergoing hip arthroscopy suggests that the LT experiences increased stress in the presence of FAIS and micro-instability [1]. The risk for LT injury in those who engage in activities that required motion beyond a point of contact between the femoral neck and acetabulum is supported by cadaveric evidence [21]. The current study suggests that the LT may play a role in maintaining stability, especially movements which involve hip external rotation combined with flexion.

The primary purpose of this research was not to define absolute stress values but add to the proof of concept in that the LT is under strain during hip movements and particularly movements in the transverse and frontal planes. Absolute stress values will largely be dependent on an individual’s specific anatomy. Characteristics which may affect the absolute stress on the LT include orientation and size of the LT itself, shape and orientation of the femoral head and acetabulum, and capsular volume with considerations for ligament laxity. These anatomical variations were intentionally omitted in this FEA model to isolate the role of the LT. Most notably, the stress experienced by the LT during external rotation, abduction, and abduction may be offset by normal intact iliofemoral, pubofemoral, and ischiofemoral ligaments, respectively. Future FEA models should account for variations in femoral version, total acetabular volume, inferior acetabulum horn morphology, and capsular characteristics to better understand how these variations may influence stress in the LT. This FEA model of the LT may serve as a template for future studies exploring these biomechanical relationships.

### Limitations

The FEA model used in this study has some limitations and may limit the generalizability of the findings. This model was built off a single MRI. While the LT was considered normal in this individual, variations exist and may impact overall LT stress. These results were obtained without accounting for the presence of a hip joint capsule, which excludes the influence of other ligaments on hip external rotation. Additionally, the FEA model assumes a spherical center for femoral head rotation, differing from actual human hip joint movements, which are influenced by torque generated by surrounding muscles. This torque can cause slight separation between the femoral head and acetabulum, potentially leading to greater stress on the LT *in vivo* compared to the FEA model’s predictions.

## Conclusion

Stress in LT was found to increase more with frontal and transverse plane movements when compared to sagittal plane movements. Therefore, the ball-and-string model with the LT functioning as a sling to wrapping around the femoral and tightening adding stability to the hip was potentially supported. Furthermore, the increase in stress with external rotation in great degrees of hip flexion suggests an increased role for the LT in hip stability as hip flexion increases. This study provides insights for understanding the LT’s contribution to hip joint mechanics.

## Supplementary Material

hnaf017_Supp

## Data Availability

The datasets used during the current study are available from the corresponding author on reasonable request.
